# Freestanding and flexible graphene papers as bioelectrochemical cathode for selective and efficient CO_2_ conversion

**DOI:** 10.1038/s41598-017-09841-7

**Published:** 2017-08-22

**Authors:** Nabin Aryal, Arnab Halder, Minwei Zhang, Patrick R. Whelan, Pier-Luc Tremblay, Qijin Chi, Tian Zhang

**Affiliations:** 1 The Novo Nordisk Foundation Center for Biosustainability, Technical University of Denmark, Kgs. Lyngby, Denmark; 2 Department of Chemistry, Technical University of Denmark, Kemitorvet, 2800 Kgs, Lyngby Denmark; 3 DTU Nanotech, Technical University of Denmark, Ørsteds Plads 345 C, DK-2800 Kongens, Lyngby Denmark; 4 School of Chemistry, Chemical Engineering and Life Science, Wuhan University of Technology, Wuhan, 430070 PR China

## Abstract

During microbial electrosynthesis (MES) driven CO_2_ reduction, cathode plays a vital role by donating electrons to microbe. Here, we exploited the advantage of reduced graphene oxide (RGO) paper as novel cathode material to enhance electron transfer between the cathode and microbe, which in turn facilitated CO_2_ reduction. The acetate production rate of *Sporomusa ovata*-driven MES reactors was 168.5 ± 22.4 mmol m^−2^ d^−1^ with RGO paper cathodes poised at −690 mV versus standard hydrogen electrode. This rate was approximately 8 fold faster than for carbon paper electrodes of the same dimension. The current density with RGO paper cathodes of 2580 ± 540 mA m^−2^ was increased 7 fold compared to carbon paper cathodes. This also corresponded to a better cathodic current response on their cyclic voltammetric curves. The coulombic efficiency for the electrons conversion into acetate was 90.7 ± 9.3% with RGO paper cathodes and 83.8 ± 4.2% with carbon paper cathodes, respectively. Furthermore, more intensive cell attachment was observed on RGO paper electrodes than on carbon paper electrodes with confocal laser scanning microscopy and scanning electron microscopy. These results highlight the potential of RGO paper as a promising cathode for MES from CO_2_.

## Introduction

Thin paper made of reduced graphene oxide (RGO) has a wide range of potential applications in research fields such as materials science, life sciences, environmental engineering, and electrochemical technologies^1–6^. RGO paper attracts interest because of its unique combination of physicochemical properties, which includes large surface area, tough mechanical strength, good biocompatibility, low cost as well as high flexibility, thermal stability, and electrical conductivity^1, 7^. In recent years, RGO papers have been explored to fabricate freestanding electrodes for electrochemical sensor applications. For instance, RGO-paper-based electrodes have been used for the detection of H_2_O_2_, glucose level in blood, pathogenic bacteria, or to monitor the secretion of nitric oxide by live cells^1, 8–10^.

Carbon paper is another paper-like material employed in electrochemical devices that share several properties with RGO papers such as high electrical conductivity, large surface area, biocompatibility, and low cost^11^. However, carbon paper has a lower mechanical strength than RGO paper due to its brittleness^7, 12^. Carbon paper has been used extensively in the field of bioelectrochemistry to fabricate electrodes for the bioelectrochemical generation of electrical energy via microbial fuel cells^13–16^. Carbon paper electrodes have also been employed in other bioelectrochemical devices such as bioelectric sensors and microbial electrolysis cells^17–19^.

Microbial electrosynthesis (MES) is a promising bioelectrochemical application in which the greenhouse gas CO_2_ is reduced into multicarbon products or methane with electrons derived from the cathode of an electrochemical reactor^20–23^. Until now, multicarbon compounds generated from CO_2_ by MES include acetate, butyrate, 2-oxobutyrate, and biofuels^24–26^. MES can be powered by electricity surpluses from the power grid as well as be partially driven by the biological oxidation of wastewater at the anode^24, 27–30^. MES reactors can also be integrated into bioinorganic artificial photosynthesis devices aiming at reducing CO_2_ with solar energy more efficiently than natural photosynthesis^20, 31–33^.

To enhance electron transfer rate and productivity of MES reactors, many efforts have focused on the development of high-performance cathode materials, microbial catalysts and growth media^34–43^. To construct an efficient cathode, the ideal material should possess good biocompatibility, high surface area, high durability, low production cost and high electrical conductivity^44^. RGO paper and carbon paper have these favorable characteristics, and thus they are used here as cathodes in a MES system with the acetogen *Sporomusa ovata* as the microbial catalyst for the present work. Furthermore, we have investigated the biofilm formation on both RGO and carbon paper electrodes and their electrochemical behavior in detail.

## Results and Discussion

### Electrode Morphology

RGO paper tested in MES reactor during this study was fabricated from high-quality GO prepared via the modified Hummer’s method^13^. UV-vis spectrum of GO showed characteristic peaks at 230 nm due to π-π* transitions of aromatic C-C bonds and a shoulder peak at ca. 300 nm due to n-π* transitions of C=O bonds (Fig. S1). To synthesize highly-conductive RGO paper, GO paper was first assembled and then reduced with a hydrazine solution and heating process. X-ray photoelectron spectroscopy (XPS) indicated that the reduction of GO paper into RGO paper was successful (Fig. S2). The XPS survey spectrum of RGO paper showed a higher C:O ratio than that for GO paper. Furthermore, a supplementary peak corresponding to nitrogen from the hydrazine reduction was observed at ca. 402 eV. The high resolution C1s spectrum from GO paper showed three different peaks centered at 284.2, 286.4, and 288.3 eV, corresponding to C-C in aromatic rings, C-O-C (epoxy and alkoxy), and O-C=O groups, respectively. As expected, the intensity of all peaks corresponding to carbon-oxygen groups, especially the C-O-C peak, decreased significantly for RGO paper, revealing that most oxygen-containing functional groups were removed during the reduction reaction. As expected, the intensity of all peaks corresponding to carbon-oxygen groups, especially the C-O-C peak, decreased significantly for RGO paper, revealing that most oxygen-containing functional groups were removed during the reduction reaction.

Raman spectroscopic measurements of GO and RGO paper (Fig. S3) showed that the apparent G peak position shifted by 20 cm^−1^ after reduction. If the apparent G peak is viewed as a superposition of G and D’ modes, then the redshift after reduction corresponds to an increase in the G peak intensity and a decrease in the D’ peak intensity^45^. The defect-related D/G peak intensity ratio^46^ is therefore decreased, which together with the decrease in D’ peak intensity are signs of increased graphitization after reduction. This is in good agreement with the results obtained from XPS. Additionally, the Raman measurement showed that the carbon paper consists of graphitic carbon (Fig. S3)^47^.

**Figure 1:**
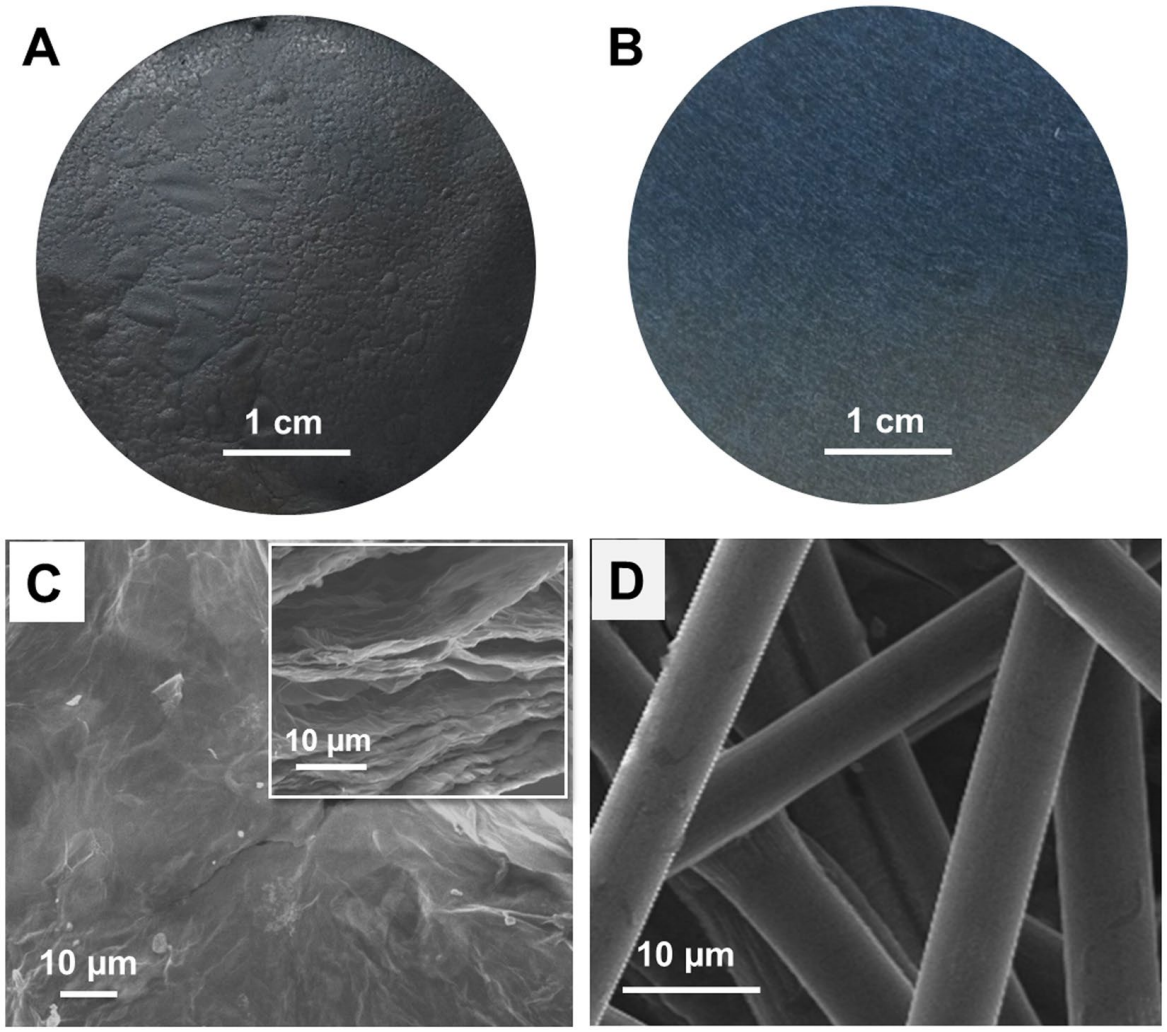
Digital pictures and SEM images of (**A**,**C**) a freestanding RGO paper cathode and (**B**,**D**) a freestanding carbon paper cathode. Inset of panel C is a cross-sectional SEM image of RGO paper.

During the fabrication process, the thickness of RGO paper was controlled at 0.36 ± 0.02 mm. This is comparable to the 0.37 mm thickness of commercially available carbon paper (AvCarb MGL370). The diameter of 4 cm was also the same for both types of cathode (Fig. 1A and B). Both carbon paper and RGO paper tested here were freestanding and are known to have high electrical conductivity^7, 48^. The specific surface area measured by the BET method of the RGO paper cathode was 0.29 m^2^ g^−1^, which is 5.4 fold higher than the carbon paper cathode with similar diameter and thickness.

Furthermore, contrary to carbon paper, RGO paper can be bent or rolled exhibiting a higher flexibility (Fig. S4). All these characteristics of RGO paper make it a good cathode candidate for MES and could be exploited to increase substantially the electrode surface area available in the MES reactor for electronic interactions with microbes. In recent studies, performance improvement observed for MES reactors equipped with carbon felt or carbon cloth cathodes coated with unfunctionalized or functionalized RGO has been attributed in part to higher specific surface area and electrical conductivity compared to unmodified cathodes^34, 35^.

### Acetate electrosynthesis from CO_2_ and current densities

**Figure 2:**
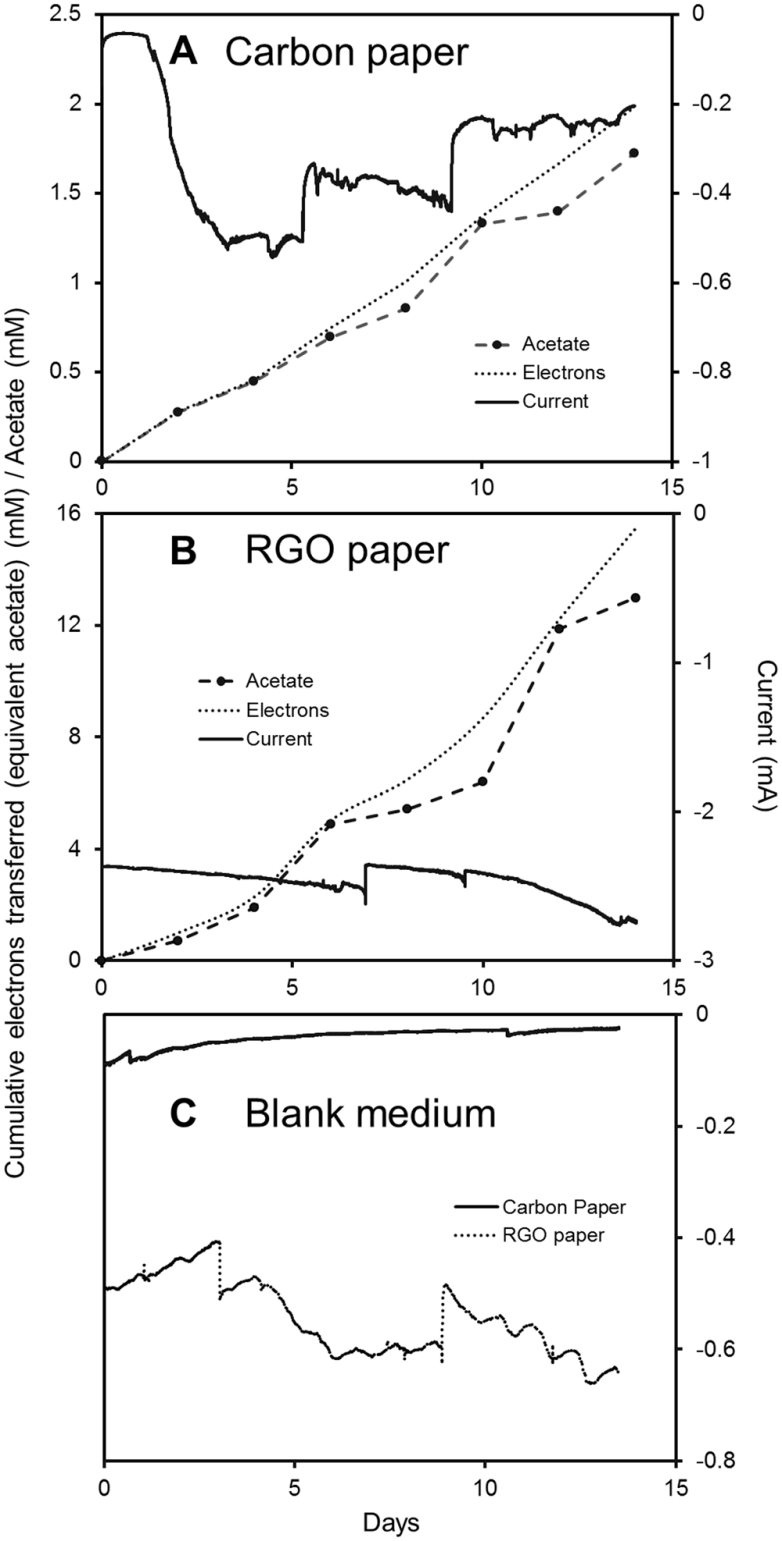
Acetate concentration, electron transferred and current consumption during MES with (**A**) a carbon paper cathode or (**B**) a RGO paper cathode. (**C**) Current consumption with carbon paper cathode or RGO paper cathode in sterile medium. The acetate concentration in mM corresponds to the analytical concentration of acetate in the reactor measured from high-pressure liquid chromatography (HPLC). Electron transferred curves measured from potentiostat refer to the acetate concentration in mM if all the electrons transferred were converted to acetate in the system. Results shown are from a representative example of three replicate bioelectrochemical reactors.

With a carbon paper cathode poised at −690 mV versus standard hydrogen electrode (SHE), the acetate production rate from CO_2_ of *S. ovata*-driven MES reactors was 20.9 ± 5.0 mmol m^−2^ d^−1^ (Fig. 2A; Table 1). When a RGO paper cathode poised at the same potential was employed, the acetate production rate was increased to 168.5 ± 22.4 mmol m^−2^ d^−1^, which is approximately an 8 fold enhancement. Furthermore, the CO_2_ conversion is highly selective, as there were no multicarbon compounds other than acetate produced from CO_2_ in noticeable quantity in this study. The current density of 2580 ± 540 mA m^−2^ was 7 fold higher with the RGO paper cathode compared to the carbon paper cathode (Fig. 2B; Table 1). Coulombic efficiency for the conversion of electrons into acetate was 90.7 ± 9.3% for RGO paper cathode and 83.8 ± 4.2% for carbon paper cathode, respectively (Table 1). The improved MES performance with RGO paper cathode could be attributed to the higher specific surface area and better biocompatibility of RGO papers.

**Table 1:** Comparison of MES performances with different freestanding carbon-based cathodes under similar operating conditions^a^.

Cathode	Microbial catalyst^b^	Production rate^c^ (mmol m^−2^ d^−1^)	Current density^c^ (mA m^−2^)	Coulombic efficiency^c^ (%)	Reference
RGO paper	*S. ovata*	168.5 ± 22.4	2580 ± 540	90.7 ± 9.3	This work
Carbon paper	*S. ovata*	20.9 ± 5.0	370 ± 100	83.8 ± 4.2	This work
RGO paper	Sterile	N.D.^d^	434 ± 54	N.A.^e^	This work
Carbon paper	Sterile	N.D.^d^	33 ± 12	N.A.^e^	This work
Carbon cloth	*S. ovata*	22.0 ± 2.0	191 ± 10	82.0 ± 3.0	34
Carbon felt	*S. ovata*	34.1 ± 10.9	400 ± 10	76.6 ± 2.3	35
Graphite stick	*S. ovata*	33.4 ± 10.8	320 ± 98	85.3 ± 8.3	36

^a^MES at a cathode potential of −690 mV vs SHE. ^b^ *S. ovata* is the wild type strain DSM-2662.

^c^Each value is the mean and standard deviation of three replicates. ^d^Not detected. ^e^Not applicable.

In the absence of *S. ovata*, MES reactors with either carbon or RGO paper cathode did not generate any acetate from CO_2_ confirming that the MES processes observed in this study were biologically-driven (Fig. 2C). Furthermore, current densities were significantly lower for abiotic MES reactors compared to MES reactors colonized by *S. ovata* for both materials (Table 1). Interestingly, the current density of abiotic MES reactors equipped with RGO paper cathode was 434.4 ± 53.8 mA m^−2^, which was 13.4-fold higher than abiotic MES reactor equipped with carbon paper cathode (Table 1). This superior current density observed with the abiotic RGO paper-equipped MES reactors is probably related to the higher specific surface area and the outstanding electrical conductivity of this material.

### Cell attachment to cathode surface

**Figure 3:**
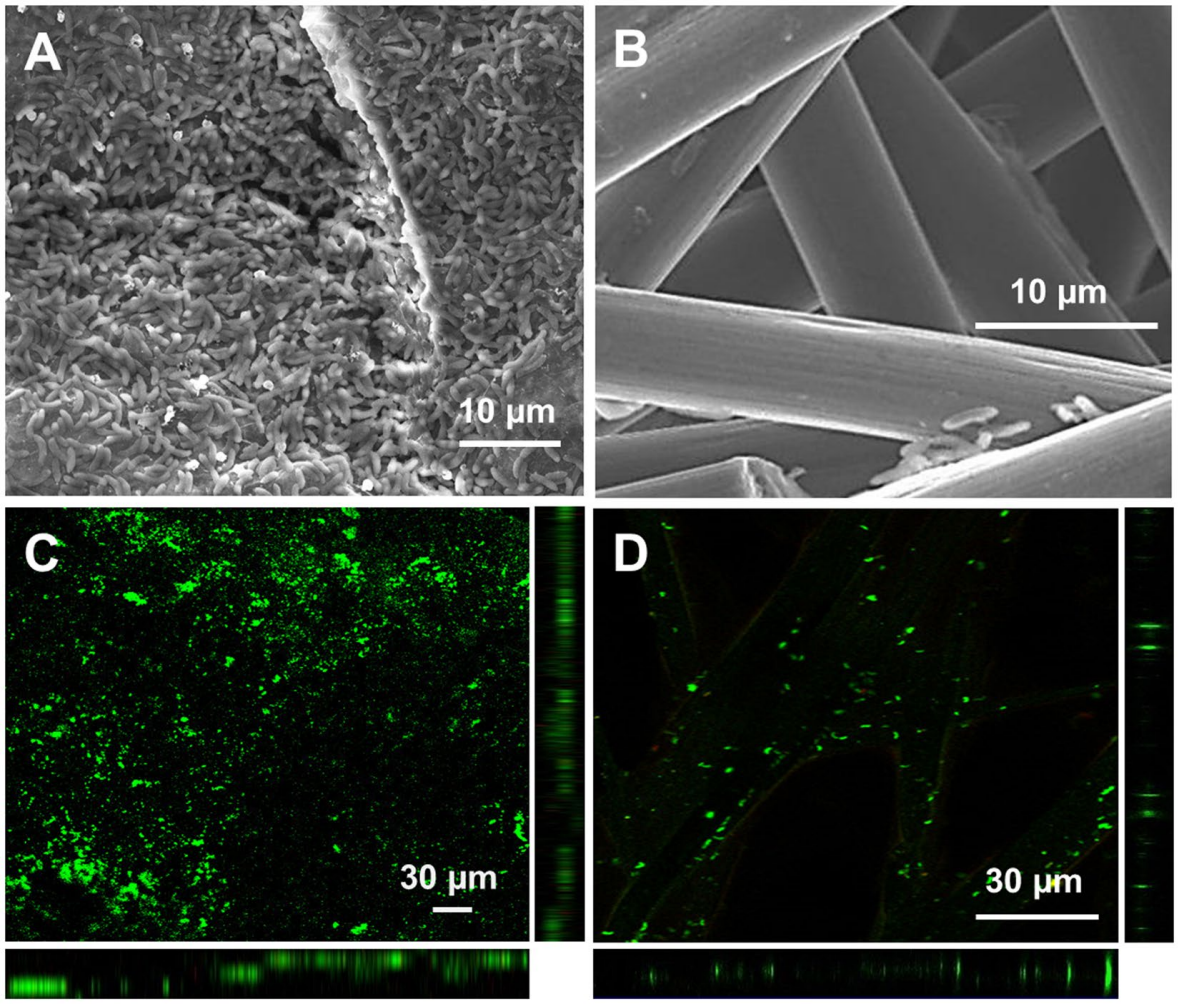
SEM and CLSM images of (**A**,**C**) a freestanding RGO paper cathode and (**B**,**D**) a carbon paper cathode in a *S. ovata*-driven MES reactor.

SEM image of RGO paper cathode from a MES system colonized by *S. ovata* and running for 12 days showed a dense biofilm composed of tightly packed bacterial cells (Fig. 3A). In comparison, only scarce and isolated bacterial cells can be observed on the SEM image of the carbon paper cathode coming from a *S. ovata*-driven MES reactor (Fig. 3B). Confocal laser scanning microscopy (CLSM) images confirmed that a more substantial *S. ovata* biofilm was formed on the RGO paper cathode than on the carbon paper cathode (Fig. 3C and D). The larger number of bacterial cells present at the surface of the RGO paper cathode indicated that this material is more compatible for colonization by S. ovata in MES reactors, which led to faster acetate production and higher current density.

### Cyclic voltammetry

**Figure 4:**
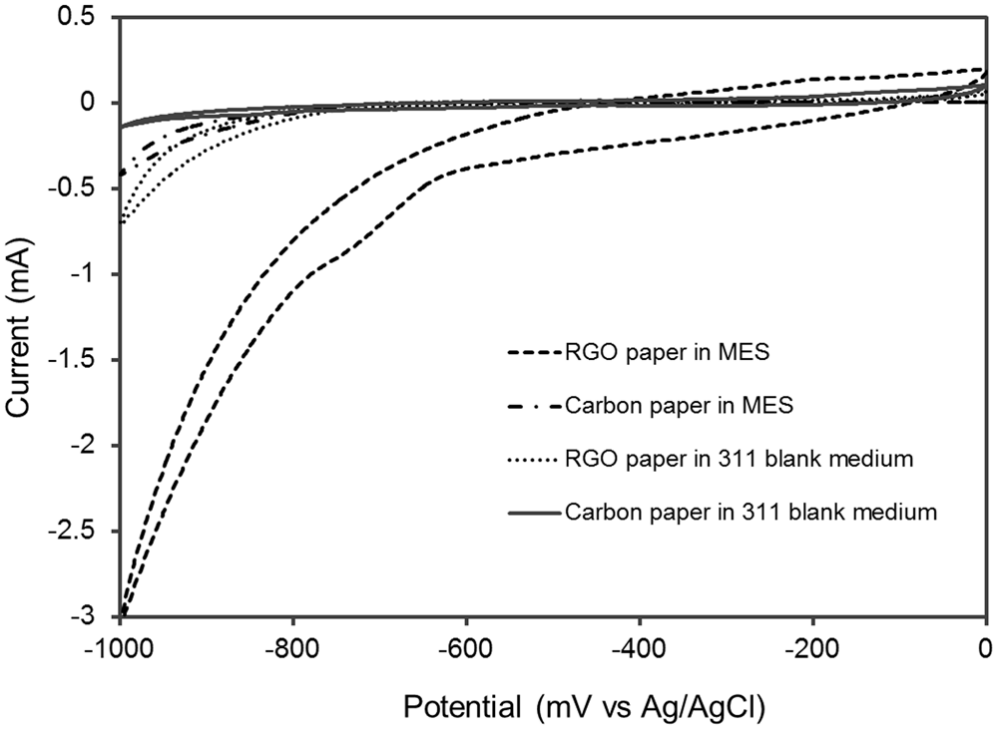
Cyclic voltammograms obtained at a RGO paper cathode or at a carbon paper cathode in *S. ovata*-driven MES reactor and in sterile blank medium. The potential window was set between −1.0 V to 0 V versus Ag/AgCl at a scan rate of 1 mV s^−1^.

The electrochemical behavior of RGO and carbon paper cathode before and after colonization by *S. ovata* during MES was investigated with cyclic voltammetry (CV) (Fig. 4). There was no reversible redox peaks detected on the current–potential curves for all the tested conditions, indicating that no electroactive species were acting as electron shuttles between *S. ovata* and the cathodes. Furthermore, in the presence of a biofilm, the RGO paper cathode exhibited a cathodic current response at −900 mV versus Ag/AgCl ca. 8 fold and 6.7 fold higher than the colonized carbon paper cathode and the sterile control RGO paper cathode, respectively (Fig. 4). Additionally, the RGO paper cathode with sterile blank medium also exhibited a higher cathodic current response than either the carbon paper cathode with a biofilm or with sterile blank medium. These results correlated well with what has been observed with the current densities of abiotic or *S. ovata*-driven MES reactors equipped with either a RGO or a carbon paper cathode.

## Conclusions

In conclusion, with wild type *S. ovata* as the microbial catalyst and at a cathode potential of −690 mV versus SHE, MES system equipped with RGO paper cathode is more performant than MES systems equipped with other types of freestanding carbonaceous cathode including carbon paper (Table 1). Besides higher acetate production rate and current density (Table 1), RGO paper cathode also has better flexibility that make it possible to be folded in the MES reactor to increase its surface area and thus improve electron exchange with microbial catalysts. Further experiments are warranted to find novel RGO paper electrode conformation that may increase the productivity of MES towards practical application.

## Materials and Methods

### The microbial catalyst *Sporomusa ovata*

*S. ovata* DSM 2662 wild type strain was acquired from the Deutsche Sammlung Mikroorganismen und Zellkulturen (DSMZ)^49^. *S. ovata* cultures were routinely maintained in the 311 medium with 40 mM betaine as substrate under a N_2_-CO_2_ (80:20) atmosphere. For growth with H_2_ as the electron source and CO_2_ as the carbon source, no betaine was added to the 311 medium and the atmosphere was N_2_-CO_2_-H_2_ (83:10:7). For all growth conditions presented in this study, casitone, sodium sulfide, yeast extract, and resazurin were omitted from the 311 medium. For MES experiments, cysteine was also omitted from the 311 medium.

### Construction of freestanding reduced graphene oxide (RGO) paper cathode

High quality graphene oxide (GO) was synthesized by the modified Hummer’s method as previously reported^35, 50^. GO solution was then used for the fabrication of RGO paper as described before^1^. Briefly, 50 ml of a 1 mg/ml GO solution was sonicated for 15 minutes before being filtered with a vacuum filtration system. Then, two-dimensional GO nanosheets were assembled into a flexible layer-by-layer GO paper structure directly on the filter membrane. The thickness and layered structural orientation of the GO paper was tuned by adjusting the volume and the concentration of the GO solution added to the vacuum filtration system. Subsequently, the GO paper was reduced to obtain highly conductive RGO paper. To this end, the GO paper was put in an autoclave container along with a few drops of hydrazine solution and heated at 180 °C overnight. After cooling to room temperature, RGO paper was washed with Milli-Q water several times and annealed further at 200 °C to remove residual hydrazine as well as to enhance its electrical conductivity. After reduction and annealing, the amount of oxygen-containing groups was significantly reduced, as evidenced by the increased C/O ratio measured by X-ray photoelectron spectroscopy (XPS). The resulting RGO paper was cut into discs with a diameter of 4 cm as cathodes.

### Microbial electrosynthesis of acetate and cyclic voltammetry

RGO paper and AvCarb carbon paper MGL 370 (AvCarb Material Solutions, USA) cathodes were tested at least in triplicate in three-electrode, dual chambered bioelectrochemical reactors. MES experiments were conducted at 25 °C with *S. ovata* DSM-2662 grown in the cathode chamber performing CO_2_ reduction to acetate as previously described^35–37, 43^. The RGO paper or carbon paper cathode (4 cm diameter, 0.36–0.37 mm thickness) and graphite stick anode (36 cm^2^) were immerged in 250 ml of 311 medium in two chambers separated by a Nafion 115 ion-exchange membrane (Ion Power, Inc., New Castle, DE, USA). The reference electrode was a sealed miniature Ag/AgCl electrode model ET072 (eDAQ, Denmark) and the cathode potential was set at −690 mV versus SHE during MES. H_2_-grown *S. ovata* cultures were established in the cathode chamber with a hydrogen-containing gas mix N_2_-CO_2_-H_2_ (83:10:7). The gas mix was switched to N_2_-CO_2_ (80:20) after several fresh medium swaps at which point data start being collected. During the whole MES experiment, the anode chamber was bubbled with N_2_-CO_2_. A CH Instrument potentiostat (CH Instruments, Inc, USA) was used to perform both MES as well as cyclic voltammetry (CV) experiments. During the CV experiments, the tested electrodes were scanned at a rate of 1 mV s^−1^ in a potential window of 0 to −1000 mV vs Ag/AgCl. Data generated during MES and CV experiments were analyzed with EC-Lab® software v.10.40 as described previously^51, 52^.

### High-pressure liquid chromatography

Acetate concentration were measured by high-performance liquid chromatography (HPLC) with an apparatus equipped with a HPX-87H anion exchange column (Bio-Rad Laboratories Inc., California, USA) at a temperature of 30 °C. The mobile phase was 5 mM H_2_SO_4_ at a flow rate of 0.6 ml/min. Refractive index detector was used for the detection and data were analyzed with the Chromeleon software (Thermo Fisher Scientific, Denmark). Where indicated, acetate production rate was normalized with respect to the projected area of RGO paper or carbon paper cathodes.

### Microscopy

For Scanning Electron Microscopy (SEM) images, cathode samples were collected and fixed during 5 hours at room temperature with a 0.1 M phosphate buffer at pH 7.0 containing 2.5% glutaraldehyde. Samples were then washed with the buffer solution without glutaraldehyde before being immerged successively in different concentration of ethanol and acetonitrile as described previously. Nitrogen-dried samples were observed with a Quanta 200 FEG scanning electron microscope (FEI) at an accelerating voltage of 10 kV under high vacuum condition. For Confocal Laser Scanning Microscopy (CLSM) images, RGO paper and carbon paper biocathodes were removed from the MES reactor and were stained with the LIVE/DEAD® BacLight™ Bacterial Viability Kit (ThermoFisher Scientific) as described previously^43^. CLSM image were taken with a Zeiss LSM 5 Pascal microscope and analyzed with the ZEN imaging software (Zeiss, Germany).

### Analytical methods

Specific surface area of RGO paper or carbon paper cathodes was determined with the Brunauer–Emmett–Teller (BET) method as previously described^53^. The Agilent 8453 G1103A spectrophotometer (Agilent, Denmark) was used to measure the UV-vis spectrum of the GO solution. X-ray photoelectron spectroscopy (XPS) was performed with a Thermo Scientific™ K-Alpha™ + X-ray Photoelectron Spectrometer System with an aluminum K-Alpha (1486 eV) as x-ray source. All the samples were deposited on polished Si-wafer by drop casting for XPS measurements. X-ray spot area for measurement was set at 400 μm and flood gun was used for charge compensation. Raman spectroscopy was conducted with a Thermo Scientific DXR Raman spectrometer equipped with a 455 nm laser.

## Electronic supplementary material

Supplementary information
