# Supplementary material for: Freestanding and flexible graphene papers as bioelectrochemical cathode for selective and efficient CO2 conversion
Source: Sci Rep. 2017 Aug 22;7:9107. doi: 10.1038/s41598-017-09841-7 (PMC5567247; doi:10.1038/s41598-017-09841-7)
Supplement: Supplementary file 1 — Supplementary information [file 41598_2017_9841_MOESM1_ESM.pdf]

## *Supplementary information*

**TITLE**

Freestanding and flexible graphene papers as bioelectrochemical cathode for selective and efficient $CO_2$ conversion.

**AUTHORS**

Nabin Aryal[1], Arnab Halder[2], Minwei Zhang[2], Patrick R. Whelan[3], Pier-Luc Tremblay[1,4], Qijin Chi[2], Tian Zhang[1,4]*

[1]The Novo Nordisk Foundation Center for Biosustainability, Technical University of Denmark, Kgs. Lyngby, Denmark

[2]Department of Chemistry, Technical University of Denmark, Kemitorvet, 2800, Kgs. Lyngby, Denmark

[3]DTU Nanotech, Technical University of Denmark, Ørsteds Plads 345C, DK-2800 Kongens Lyngby, Denmark

[4]School of Chemistry, Chemical Engineering and Life Science, Wuhan University of Technology, Wuhan 430070, PR China

*Corresponding author: zhang@biosustain.dtu.dk

**Supporting data**

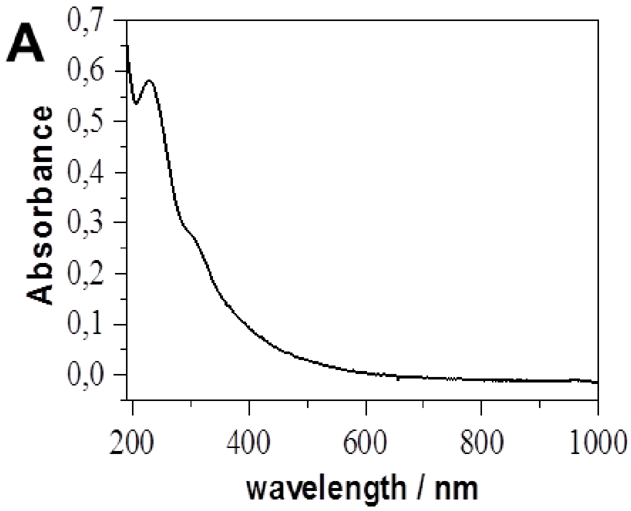


**Figure S1.** UV-vis spectrum of GO.

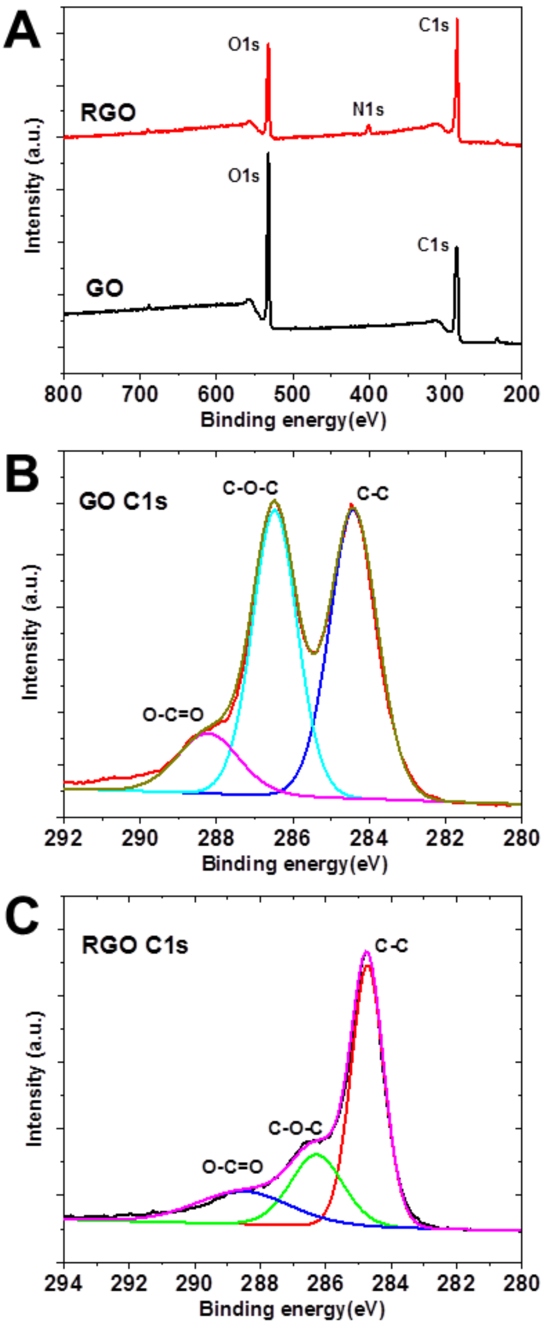


**Figure S2.** (A) The XPS survey spectra for GO and RGO. The high resolution C 1s spectra for (B) GO and (C) RGO.

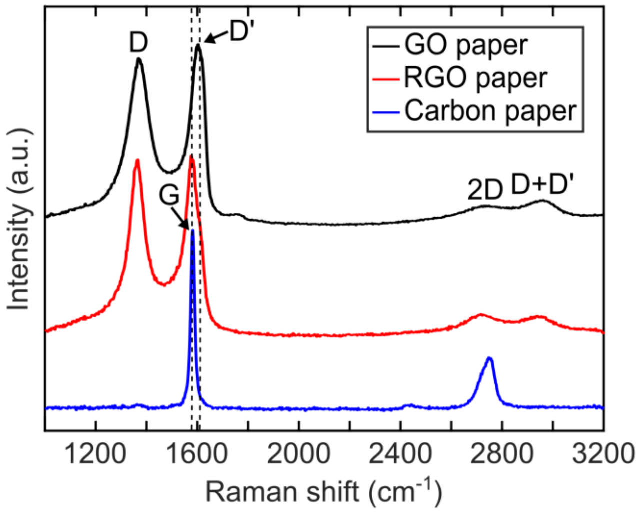


**Figure S3.** Raman spectra of GO, RGO and carbon paper.

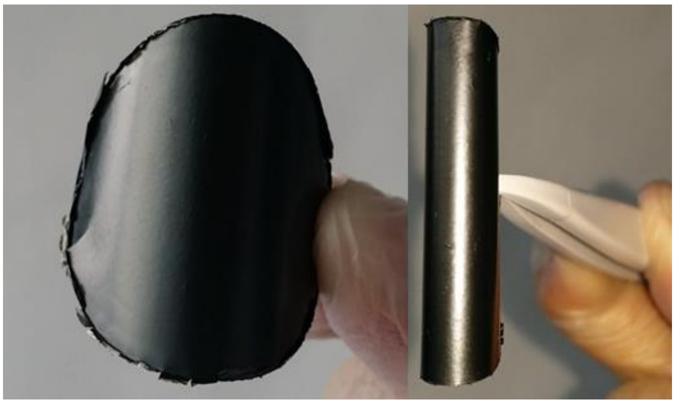

**Figure S4.** A bent RGO paper for demonstrating its mechanical flexibility.
